# Study on the Cutting Damage Mechanism of Aramid Honeycomb Based on the Progressive Damage Model

**DOI:** 10.3390/ma15124063

**Published:** 2022-06-08

**Authors:** Yuxing Yang, Yongjie Bao, Jinlong Wang, Chen Chen

**Affiliations:** 1 Marine Engineering College, Dalian Maritime University, Dalian 116026, China; yangyuxing@dlmu.edu.cn (Y.Y.); wjl19890806@dlmu.edu.cn (J.W.); 2 Naval Architecture and Ocean Engineering College, Dalian Maritime University, Dalian 116026, China; chen_chen@dlmu.edu.cn

**Keywords:** aramid honeycomb, cutting damages, cutting process, finite element modelling

## Abstract

A progressive damage model for aramid honeycomb cutting was proposed to reveal its cutting damage mechanism. It established the relationship between the mesoscale failure modes and the macroscale cutting damage types of the aramid honeycomb. The proposed model addressed the material assignment problem of impregnated honeycomb by developing a material calculation method that simulates the real manufacturing process of the aramid honeycomb. Cutting experiment of aramid honeycomb specimen was conducted concerning on the cutting forces response and cutting damages, which validated that the proposed method was effective for investigating the cutting process and mechanism for the aramid honeycomb. Predicted cutting mechanism results show that: (a) cutting process of the aramid honeycomb can be divided into three stages with four characteristic states—initial state, cut-in state, cut-out state and final state; (b) cell wall bending in the cutting direction relieves the cutting force, and strong plasticity of the aramid fiber makes it hard to break, which lead to uncut fiber and burr damages; (c) using sharp tip cutting tool to reduce cutting force and bonding both top and bottom of the honeycomb to make it stiffer are beneficial to obtain good cutting quality with less damages.

## 1. Introduction

Aramid honeycomb, such as Nomex^®^, whose mechanical properties show orthotropic anisotropy due to the unique close-packed hexagonal structure [1], is a porous material made of aramid fiber and phenolic resin [2]. It exhibits good performance, such as high specific strength, excellent flame retardancy, good corrosion resistance, and shock absorption [3], which makes it a promising core material for lightweight collision energy absorption structures in aerospace engineering like fairing, flap and hatch door, and in marine engineering like hull of racing boat and fireproof bulkhead [4,5,6,7,8].

The aramid honeycomb is usually manufactured by the stretching expansion process, during which the shape of the aramid paper is fixed by glue [9,10]. After stabilizing, honeycomb core is dipped in the phenolic resin. Finally, it can be cut into plates with desired size [11]. Generally, the cutting process will lead to inevitable defects [12], such as tear, burr, and uncut fiber. Observable cutting defects in the experiment are limited and most of them are the final results. In order to reveal the cutting mechanism during the cutting process, it is necessary to establish numerical model of honeycomb cutting, which can help understand evolution of the cutting defects.

A good numerical model of honeycomb cutting, which has a good match with the experimental results, can visually reveal the cutting deformation and damage mechanism of the honeycomb with lower costs than experiments. A lot of numerical methods for honeycomb modelling have been developed, which can be mainly divided into two types: homogenized model and detailed model. The homogenized model, including isotropic homogenized model and orthotropic homogenized model, neglects the multi-layer nature. The isotropic homogenized model neglects orthotropic material behavior of the honeycomb material, while the orthotropic homogenized model considers it. The detailed model considers the multi-layer nature of the honeycomb material, which models the aramid paper and phenolic resin individually. Four modelling approaches of Nomex honeycomb were benchmarked in literature [13], including single-layer isotropic approach (isotropic homogenized model), single-layer orthotropic approach (orthotropic homogenized model), multi-layer resin coating approach (detailed model based on planar laminate theory), and multi-layer resin corner approach (detailed model considering resin accumulation in the hexagon corners); the single-layer orthotropic approach (orthotropic homogenized model) enabled simultaneous calibration for all four loading conditions and had a good agreement with the test results, while a detailed model respecting the real geometry of the cells can give more information [14]. In addition, an analytical homogenized model of composite cell wall honeycomb was also developed by Wang et al. [15] for computing costs reducing by modelling the locally heterogeneous honeycomb as a homogeneous orthotropic bulk, whose stiffness matrix was derived by combining Gibson and Ashby’s model [16] and the classic laminated beam theory model.

Jaafar et al. [17] reported an orthotropic finite element modelling method of the Nomex honeycomb cutting based on the 2D Hashin criteria, which predicts the cutting forces and surface quality compared with the experimental results. Furthermore, the model based on the 2D Hashin criteria was compared with that based on the Tsai-Wu criteria, and it was found that the 2D Hashin criteria will underestimate the cutting forces [14], which indicates that the out-of-plane delamination should not be neglected. Liu et al. [18] used mesoscopic shell model based on the 2D Hashin criteria, which includes fiber tensile fracture, fiber compressive fracture, matrix tensile failure, and matrix compressive failure to characterize the tearing defects of the Nomex honeycomb wall during cutting, and it was reported that the deformation of honeycomb wall and the cutting force in direction parallel to honeycomb wall have a major impact on the formation of tearing defects.

In this paper, to reveal the cutting mechanism in the process of aramid honeycomb cutting, a detailed finite element model (FEM) based on the progressive damage model using 3D Hashin-type criteria is proposed, which can predict seven types of failures. The honeycomb cutting model is established by simulating the real manufacturing process, which considers the fact that honeycomb paper is composed by the core material and the resin. As a result, mechanical properties of the impregnated aramid honeycomb can be calculated based on the series model by composing mechanical properties of the core material and that of the resin. A validation cutting experiment using disc cutting tool is conducted, whose cutting force response and damages are compared with that from the finite element model. Finally, the cutting mechanism during cutting the aramid honeycomb is revealed using the proposed FEM.

## 2. Finite Element Modelling

### 2.1. Honeycomb Cutting Model

**Figure 1:**
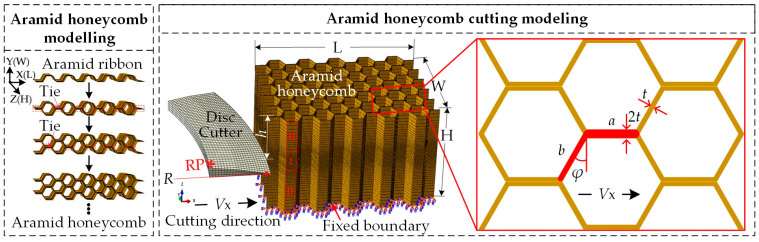
Finite element modelling of the aramid honeycomb cutting process.

In order to study the cutting mechanism of the aramid honeycomb cutting, finite element analysis was conducted by Abaqus/Explicit with VUMAT subroutine. The aramid honeycomb was modeled by simulating the real manufacturing process as shown in Figure 1. First of all, an aramid ribbon with fixed shape was modeled as shown in Figure 1 and assigned with impregnated honeycomb material, which considers both mechanical properties of the core material and that of the resin. Then two contact surfaces of the adjacent ribbons were tied together at the position of the double wall, which makes the translational and rotational motion, as well as all other active degrees of freedom equal for this pair of surfaces, simulate the interface bonding effect between ribbons. The honeycomb structure was formed when all ribbons were constrained together. The *X*-axis in Figure 1 is along the direction of ribbon length L, the *Y*-axis is along the out-of-plane direction W, and the *Z*-axis is along the direction of expansion H. The geometry of the aramid honeycomb was illustrated in Figure 1, in which L, W, and H are length, width, and height of the specimen, *h* is cutting depth, *a* is double wall length, *b* is single wall length, *t* is single wall thickness, and *φ* is corner angle.

Sharp disc cutting tool was modeled as a 3D discrete rigid body constrained to the reference point RP. The cutting tool tip was treated as an arc with radius of 0.01 mm to improve the contact effect between the cutting tool and honeycomb in the cutting process. The global mesh size of the cutting tool was 0.5% of the cutting tool diameter (about 0.5 mm), while mesh was refined at the cutting tool tip with the smallest element size of 0.01% of the cutting tool diameter. As for the honeycomb specimen, C3D8R element (3D 8-node reduced integral element in Abaqus) with ‘stiffness’ hourglass control method was used. Considering the computational cost and results accuracy, mesh size within the area I (contact area between the cutting tool and the honeycomb specimen) was set as about 0.8% of the specimen height (about 0.1 mm), while that within the area II changes from about 4% to 0.8% of the specimen height by using single direction bias (from 0.5 mm to 0.1 mm).

Constant velocity *V*_x_ along the direction of ribbon length was loaded on the reference point RP of the cutting tool. Displacement *U*_x_, reaction forces *RF*_x_ and *RF*_y_ of the reference point were recorded in FEM. As for contact conditions between the cutting tool and the specimen, ‘Hard contact’ enforced with a Lagrange multiplier representing the contact pressure in a mixed formulation was used to model the normal behavior, and Coulomb friction implemented with the penalty contact algorithm (relative motion in the absence of slip is equal to the friction force divided by the penalty stiffness) was established to model the tangential behavior. Due to discontinuous distribution and small thickness of the honeycomb walls, the contact area between the wall and the cutting tool is minimized so that a low friction coefficient between 0.1~0.25 [14,19,20] is fine for simulation.

### 2.2. Material Properties

Mechanical properties of the aramid honeycomb are determined by the core material (e.g., Nomex paper) and the resin (e.g., Phenolic resin) considering its manufacturing process. First, mechanical properties of the core material and the resin can be obtained from the manufacturer and/or research papers. Then mechanical properties of the impregnated aramid honeycomb paper can be calculated based on the superposition principle considering the fact that honeycomb paper was composed by core material and resin, as shown in Equations (1)–(6). The symbols with superscript ‘co’ and ‘re’ represent the mechanical property of the core material and mechanical property of the resin, respectively, while that without superscript represents the mechanical property of the impregnated aramid honeycomb paper. The symbol with subscript ‘T’ represents the tensile property, while that with subscript ‘C’ represents the compressive property. The subscript ‘L’ means the direction of ribbon length (longitudinal direction), while subscript ‘H’ means the direction of expansion (transverse direction).

Elastic modulus along the direction of ribbon length *E*_L_ and that along the direction of expansion *E*_H_ of the impregnated aramid honeycomb paper can be expressed as follows:

(1) $$E_{L}=E_{L}^{\mathrm{co}}(1-V^{\mathrm{re}})+E_{L}^{\mathrm{re}}V^{\mathrm{re}}$$

(2) $$E_{H}=E_{H}^{\mathrm{co}}(1-V^{\mathrm{re}})+E_{H}^{\mathrm{re}}V^{\mathrm{re}}$$

where *V*^re^ (volume of the resin) represents the resin content, which is related with the density of the aramid honeycomb, as given in Table 1.

In-plane Poisson’s ratio *v*_in_ and out-of-plane Poisson’s ratio *v*_out_ of the impregnated aramid honeycomb paper can be obtained by Equations (3) and (4).

(3) $$\nu_{\mathrm{in}}=\nu_{\mathrm{in}}^{\mathrm{co}}(1-V^{\mathrm{re}})+\nu^{\mathrm{re}}V^{\mathrm{re}}$$

(4) $$\nu_{\mathrm{out}}=\frac{\nu_{\mathrm{in}}(E_{L}-\nu_{\mathrm{in}}E_{W})}{E_{L}(1-\nu_{\mathrm{in}})}$$

Density of the impregnated aramid honeycomb paper ρ can be obtained by Equation (5).

(5) $$\rho =\rho^{\mathrm{co}}(1-V^{\mathrm{re}})+\rho^{\mathrm{re}}V^{\mathrm{re}}$$

Tensile strength along the direction of ribbon length *S*_LT_ and that along the direction of expansion *S*_HT_, compressive strength *S*_LC_ and *S*_HC_, shear strength *S*_LS_ and *S*_HS_, can be calculated by Equation (6).

(6) $$S_{ij}=S_{ij}^{\mathrm{co}}(1-V^{\mathrm{re}})+S_{ij}^{\mathrm{re}}V^{\mathrm{re}}\;\;(i=L,\;H;\;j=T,\;C,\;S)$$

### 2.3. Damages Prediction

Core material of the honeycomb has obvious anisotropic and heterogeneous characteristics. Considering the independence of fracture modes of the matrix and the core material, transversely isotropic stress–strain relationship based on the classical laminate theory and Hashin-type progressive damage model were used in the finite element model [14,22]. During the cutting procedure, disc cutting tool contacts the cell wall of the honeycomb, whose aramid fibers will be broken mainly under bending forces and resin will be crushed mainly under out-of-plane compressive forces on mesoscale. As a result, honeycomb will be cut into two parts with out-of-plane delamination damages of the cell wall on macroscale. When the cutting tool is about to cut out, cell wall of the honeycomb will have obvious tensile fracture due to weak support so that failure in tension for both aramid fibers and resin must be considered. Meanwhile, because of thickness being too thin for the cell wall, aramid fibers will be pulled out of the resin mainly by shear forces during cutting so that it is necessary to predict the fiber-matrix shear-out damage. Therefore, 3D Hashin criteria, with seven types of failures, were developed to predict the damage initiation of the honeycomb, including fiber tensile failure, fiber compressive failure, in-plane matrix cracking, in-plane matrix crushing, out-of-plane delamination between ribbons in tension, out-of-plane delamination between ribbons in compression, and fiber-matrix shear-out. Failure initiation criteria, conditions, and schemes for different failure types are given in Table 2, where *σ_i_* (*i* = 1, 2, 3) are normal stresses, *τ_ij_* (*i*, *j* = 1, 2, 3; *i* ≠ *j*) are shear stresses.

When one of the above equations satisfies that *e_k_* ≥ 1 (*k* = FT, FC, MT, MC, DT, DC, FMS), the damage variable *d_k_* will be set to one, which represents that the element has been damaged. Otherwise, *d_k_* equals zero. Then, effective stiffness matrix **C**^d^ associated with stiffness degradation after damage initiation point can be expressed by Equations (7) and (8). The subscript FF, MF, and DF represent fiber failure (tensile or compressive), in-plane matrix failure (cracking or crushing), and delamination (in tension or compression), respectively, which depend on the specific strain status. Taking fiber tensile failure as an example, *d*_FF_ is equal to *d*_FT_.

(7) $$C^{d}=\left[\begin{array}{cccccc} C_{11}^{d} & C_{12}^{d} & C_{13}^{d} & 0 & 0 & 0\\ & C_{22}^{d} & C_{23}^{d} & 0 & 0 & 0\\ & & C_{33}^{d} & 0 & 0 & 0\\ & \mathrm{symmetric} & & C_{44}^{d} & 0 & 0\\ & & & & C_{55}^{d} & 0\\ & & & & & C_{66}^{d} \end{array}\right]$$

(8) $$\begin{array}{l} C_{11}^{d}=(1-d_{\mathrm{FF}})C_{11},\;C_{22}^{d}=(1-d_{\mathrm{FF}})(1-d_{\mathrm{MF}})C_{22},C_{33}^{d}=(1-d_{\mathrm{FF}})(1-d_{\mathrm{DF}})C_{33},\\ C_{12}^{d}=(1-d_{\mathrm{FF}})(1-d_{\mathrm{MF}})C_{12},C_{13}^{d}=(1-d_{\mathrm{FF}})(1-d_{\mathrm{DF}})C_{13},C_{23}^{d}=(1-d_{\mathrm{MF}})(1-d_{\mathrm{DF}})C_{23},\;\\ C_{44}^{d}={\left(1-d_{\mathrm{FMS}}\right)}^{2}C_{44},\;C_{55}^{d}=(1-d_{\mathrm{FF}})(1-0.9d_{\mathrm{DT}})(1-0.5d_{\mathrm{DC}})C_{55},\\ C_{66}^{d}=(1-0.9d_{\mathrm{MT}})(1-0.5d_{\mathrm{MC}})(1-0.9d_{\mathrm{DT}})(1-0.5d_{\mathrm{DC}})C_{66} \end{array}$$

The abovementioned progressive damage model with failure initiation criteria and damage propagation method was coded using FORTRAN language and implemented in VUMAT subroutine program in Abaqus. Damages status for seven failure types, FT, FC, MT, MC, DT, DC, and FMS, were visualized in Abaqus post-processing as solution-dependent state variables (SDV) 1~7.

## 3. Validation Experiment

### 3.1. Specimen and Material

**Figure 2:**
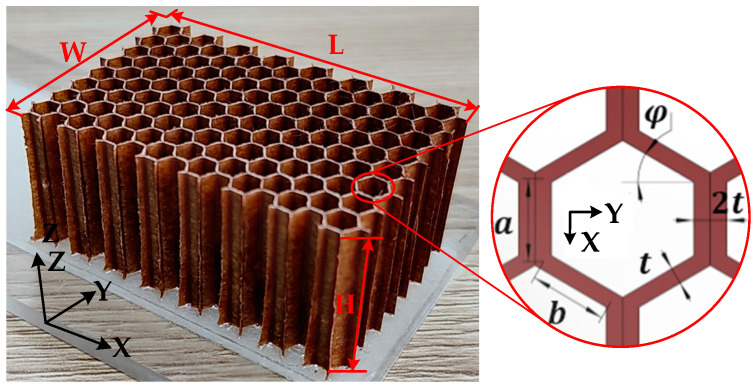
Aramid honeycomb specimen of the cutting experiment.

As shown in Figure 2, the specimen of the cutting experiment was the aramid honeycomb ACCH-1-1.83-48 with dimensions of 30 mm (L) × 20 mm (W) × 12 mm (H) manufactured by AVIC Composite Corporation, whose paper thickness was 0.05 mm and resin content was 34.1%. The single wall thickness *t* was 0.05 mm, both double wall length *a* and single wall length *b* were 1.83 mm, and corner angle φ was 30°. Material properties of the core material, resin, and the impregnated honeycomb are given in Table 3. The material properties of the impregnated honeycomb were calculated by Equations (1)–(6).

### 3.2. Experimental Set-Up

**Figure 3:**
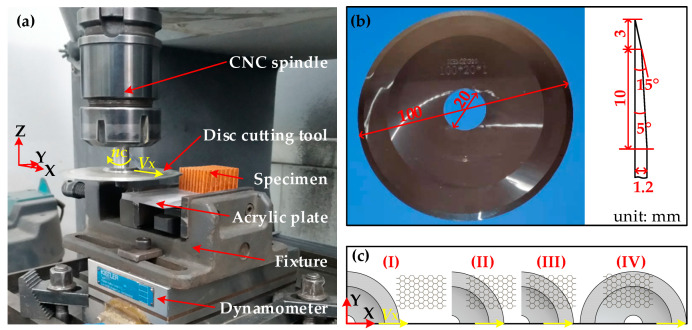
Experimental set-up and cutting process: (**a**) experimental set-up; (**b**) disc cutting tool; (**c**) cutting process of the honeycomb.

Cutting experiment of the aramid honeycomb was conducted on a three-axis CNC machine (maximum spindle speed 12,000 r/min). Figure 3a shows the experimental set-up. In order to guarantee the clamp effect during the cutting process and improve the stiffness of the honeycomb specimen, it was bonded to an acrylic plate by double sided tape, which is easily removed after cutting. Cutting forces in the X-direction and Y-direction plane were measured by a dynamometer 9257B (Kistler, Switzerland, sensitivity ≈ −7.5 pC/N, hysteresis ≤ 0.5% FSO) with acquisition frequency of 20 Hz. Charge amplifier LN5861 (Sino Ceramics, State College, PA, USA) and data acquisition USB1902 (ADLINK, Taibei, China) were used to record data from the dynamometer into a personal computer. The cutting damage size was measured by a digital microscope with super wide depth of field VHX-600E (Keyence, Japan, magnification 20×~5000×). As shown in Figure 3b, a disc cutting tool made by 9CrSi with a sharp tip was used as the cutting tool, whose diameter was 100.0 mm and maximum thickness was 1.2 mm. Figure 3c shows the cutting process: (I) at the initial state, there is no contact between the cutting tool and the specimen; (II) at the cut-in state, the contact width between the cutting tool arc and specimen equals the width of the specimen; (III) after the cut-out state, the contact width will be less than the width of the specimen; (IV) at the final state, the specimen is completely cut through. During the cutting experiment, spindle speed was *n_c_* = 5000 r/min, feed speed was *V*_x_ = 120 mm/min, cutting depth was *h* = 6 mm.

## 4. Results and Discussion

### 4.1. Validation Results

**Figure 4:**
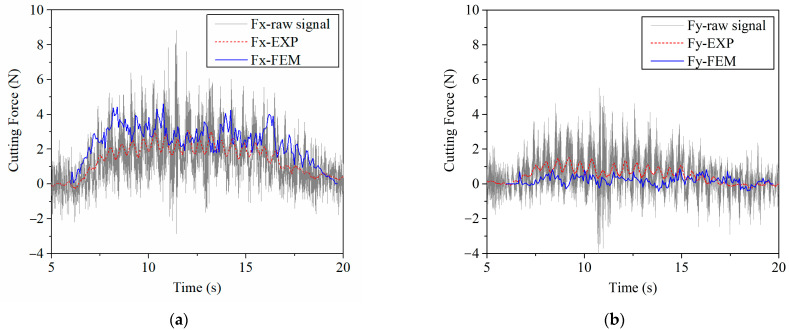
Comparison of cutting forces from experiments and finite element model: (**a**) X direction: along the cutting direction; (**b**) Y direction: transverse to the cutting direction.

During the cutting procedure using a thin disc cutting tool, the cell wall of the honeycomb specimen was broken under the thrust forces in the direction parallel to the cutting tool moving direction so that force ‘Fx’ (along the cutting direction) and ‘Fy’ (transverse to the cutting direction) were concentrated. The charge amplifier converted the measurement signals into electrical voltages, which were exactly proportional to the force acting. Then, raw force signals ‘Fx-raw signal’ and ‘Fy-raw signal’ were obtained. In order to reduce the interference of high frequency signal, the raw signals from the experiments were smoothed by the Savitzky–Golay method with second order polynomial and 150 points of windows in Origin software. As a result, the smoothed cutting force–time curves ‘Fx-EXP’ and ‘Fy-EXP’ in Figure 4 were obtained. Then, they were compared with the reaction forces of the reference point RP from the FEM, which was used as the predicted FEM values.

As can be seen in Figure 4, predicted ‘cutting force–time’ responses ‘Fx-FEM’ and ‘Fy-FEM’ were close to the results from the experiments during the whole cutting period. The average cutting forces of the experiment (smoothed curves) between the cut-in state (time 9.4 s) and the cut-out state (time 16.88 s) are Fx = 2.09 N and Fy = 0.85 N, while that of the FEM are Fx = 2.85 N and Fy = 0.24 N. Both cutting forces from FEM and from experiments were too small to compare with the relative errors. Absolute errors of Fx and Fy between the FEM and the experiment were 0.76 N and −0.61 N, respectively, which were all less than 1 N. Meanwhile, the cutting forces change greatly at the cut-in state and the cut-out state, which makes the difference between the FEM and the experiment bigger.

**Figure 5:**
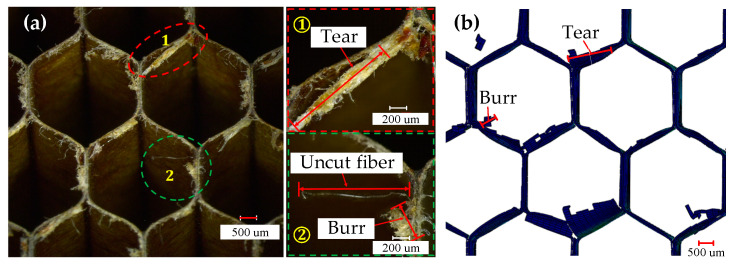
Comparison of damages from the (**a**) experiments and (**b**) FEM.

Damages of the specimen from the experiment and the FEM are shown in Figure 5. It was found that major damages in the process of cutting aramid honeycomb conclude tear, uncut fiber, and burr. Cutting force acting on the low stiffness cell wall of the honeycomb in the cutting direction makes the cell wall bent, which will relieve the cutting force, and strong plasticity of the aramid fiber (longitudinal elongation at break is 8.7% and transverse elongation at break is 5.4%) makes it hard to break. As a result, there will be uncut fiber and burr damages. Tear damage was formed due to weak support on the cell wall and bad bonding effect between fibers and resin. Both tear and burr damages in the experimental specimen can be observed in the FEM. Maximum length of the tear damage in the experiment was about 1.41 mm, while that in the FEM was about 1.53 mm with relative error of 8.5% between the FEM and the experiment. Maximum length of the burr damage in the experiment was about 0.47 mm, while that in the FEM was about 0.52 mm with relative error of 10.6% between the FEM and the experiment.

Considering the simulation error caused by the mass scaling method, material properties error between the FEM and the experiment specimen, as well as the measurement sensitivity, the predicted force Fx was acceptable for relative comparisons due to the fact that the well predicted damages were more important than the well predicted force values in the discussion section focuses on the cutting damage mechanism. Therefore, the proposed finite element method can be used to investigate the cutting process for the aramid honeycomb.

### 4.2. Cutting Mechanism

**Figure 6:**
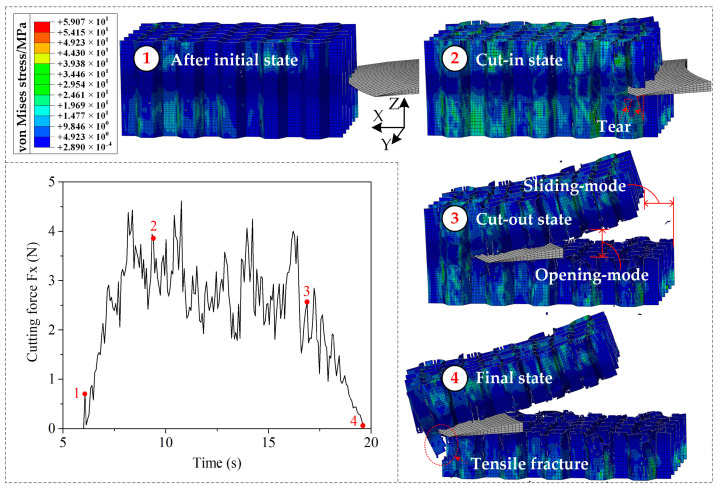
Predicted cutting process with sharp disc cutting tool by FEM.

Using the validated finite element method, the cutting mechanism of the Aramid honeycomb can be revealed visually. The cutting process with sharp disc cutting tool predicted by the FEM was shown in Figure 6. After the initial state, the cutting tool starts to contact the cell wall of the honeycomb. The cells near the cutting tool start to deform under the thrust force of the cutting tool, and it will be cut off when the fiber tensile failure criterion or fiber compressive failure criterion is met. When it comes to the cut-in state, as specified in Figure 3c, sliding-mode cracking (Mode II fracture) can be seen, which is related with the cutting tool tip geometry (discuss later). Tear damage occurs at where elements between walls of the same cell were failed, as marked by red triangles. At the cut-out state, the specimen was cut off in opening-mode cracking (Mode I fracture) and sliding-mode cracking (Mode II fracture) modes. At the final state, the specimen was completely cut through in the Mode I and Mode II mixed fracture mode with significant tensile fracture of the cell wall due to weak support.

### 4.3. Effect of the Cutting Tool Tip Geometry

**Figure 7:**
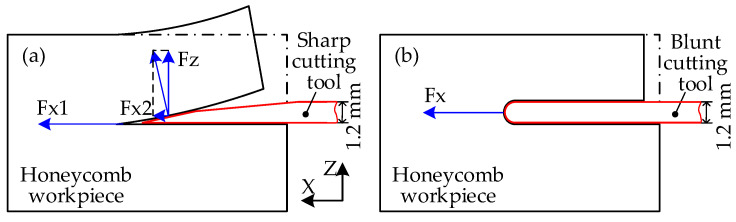
Free-body diagrams of honeycomb cutting with different cutting tools: (**a**) sharp cutting tool; (**b**) blunt cutting tool.

The cutting quality of the aramid honeycomb is strongly related to the cutting tool tip geometry. Two types of disc cutting tools were compared, sharp disc cutting tool and blunt disc cutting tool, as shown in Figure 7. Thickness of the blunt disc cutting tool was equal to the maximum thickness of the sharp disc cutting tool, which was 1.2 mm. Comparing the free-body diagrams of the honeycomb cutting with two different cutting tools, it is reasonable to predict that the honeycomb cut by the sharp disc cutting tool will fracture in the mixed-mode cracking mode, while the honeycomb cut by the blunt disc cutting tool will fracture in the sliding-mode cracking dominant mode. Meanwhile, deformation along the *X*-axis of the honeycomb cut by the blunt disc cutting tool will be greater than that cut by the sharp disc cutting tool due to the fact that thrust force along the cutting direction of the blunt tool is higher than that of the sharp tool.

**Figure 8:**
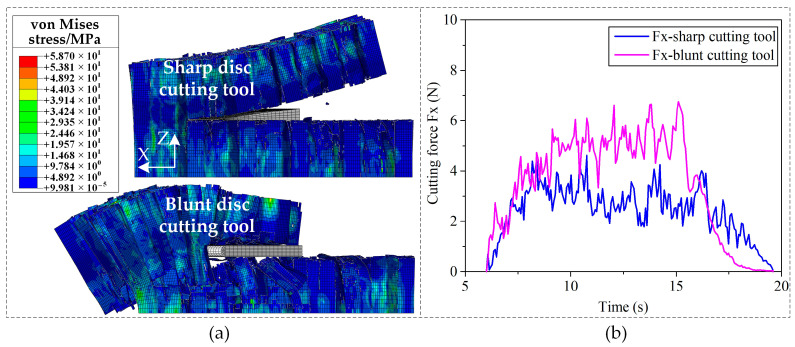
Predicted stress distributions and cutting forces with different cutting tool tip geometries: (**a**) predicted stress distributions; (**b**) predicted cutting forces.

Predicted stress distributions at the cut-out state and predicted cutting force Fx by the FEM with different cutting tool tip geometries (sharp disc cutting tool and blunt disc cutting tool) were compared in Figure 8. The honeycomb cut by the sharp disc cutting tool deforms along the *X*-axis and *Z*-axis obviously, while that cut by the blunt disc cutting tool mainly deforms along the *X*-axis. It means that the honeycomb fractures in the mixed-mode for the former, while it fractures in the sliding-mode cracking dominant mode for the latter. Deformation along the *X*-axis for honeycomb cut by the blunt disc cutting tool is much greater than that cut by the sharp disc cutting tool, which has good agreement with the force diagram results in Figure 7. Meanwhile, the honeycomb cut by the blunt disc cutting tool has been fractured before the cutting tool is completely cut out due to big thrust force of the blunt cutting tool tip, which leads to the sudden drop of the cutting force at the cut-out state (time = 16 s) as shown in Figure 8b. Therefore, using sharp tip cutting tool for aramid honeycomb cutting makes the cutting force lower, which is advantageous for obtaining good cutting quality.

### 4.4. Effect of the Specimen Boundary Conditions

**Figure 9:**
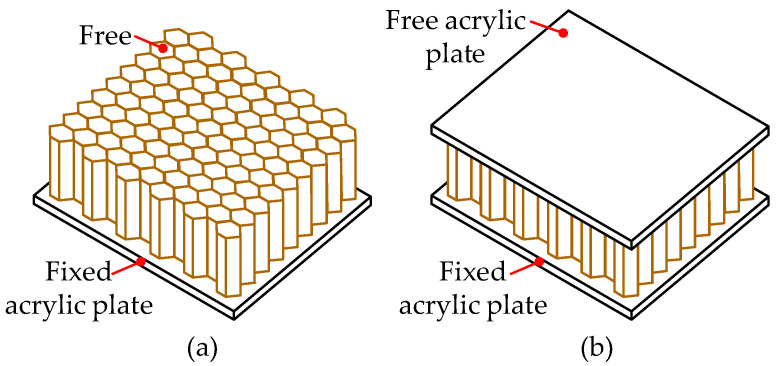
Illustration of boundary conditions for the honeycomb: (**a**) BC1; (**b**) BC2.

In order to study the effect of the specimen boundary conditions on the cutting mechanism, two different boundary conditions, BC1 and BC2, were studied. As shown in Figure 9, the boundary condition BC1 represents that top of the honeycomb is free, while the BC2 represents that top of the honeycomb is bonded to an acrylic plate. It should be noted that the free acrylic plate on the top of the honeycomb was represented by rigid body constraint in Abaqus, in which the topmost nodes of the specimen were tied to a reference point as a rigid body to simulate the acrylic plate. The bottom of the honeycomb is fixed in the FEM for both boundary conditions BC1 and BC2.

**Figure 10:**
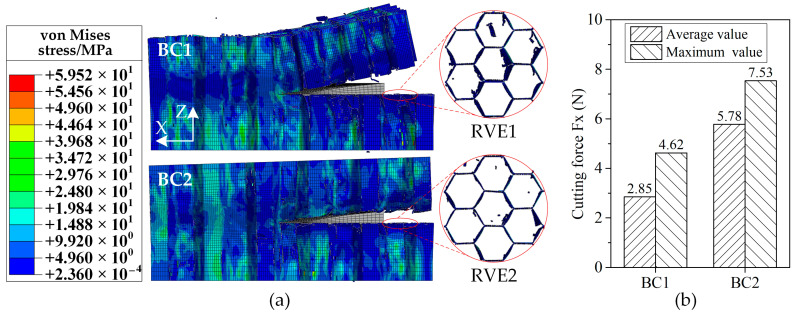
Predicted stress distributions and cutting forces under different specimen boundary conditions: (**a**) predicted stress distributions; (**b**) predicted cutting forces.

**Figure 11:**
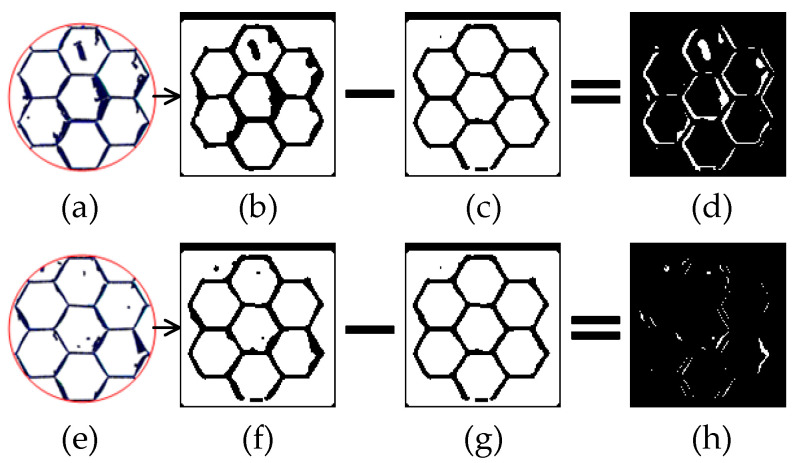
Calculating method of damage areas for two BC configurations: (**a**) damaged RVE1 with BC1; (**b**) greyscale image of the RVE1; (**c**) undamaged image of the RVE1; (**d**) damaged area of the RVE1; (**e**) damaged RVE2 with BC2; (**f**) greyscale image of the RVE2; (**g**) undamaged image of the RVE2; (**h**) damaged area of the RVE2.

The contours of von Mises stress for the aramid honeycomb cutting with boundary conditions BC1 and BC2 at the same cutting length along the *X*-axis are shown in Figure 10. It can be seen that deformation of the top part of the honeycomb at the *Z*-axis for the BC1 configuration is obviously greater than that for the BC2 configuration because the rigid body constraint limits the bending deformation on the top of the honeycomb. As a result, sliding-mode cracking (Mode II fracture) dominates the honeycomb cutting process for the BC2 configuration, while opening-mode cracking (Mode I fracture) is less important, which results in the cutting force Fx of the BC2 configuration being bigger than that of the BC1 configuration in Figure 10b. In order to compare the damages under different boundary conditions, representative volume elements (RVE) with seven honeycomb cells (as named RVE1 and RVE2 in Figure 10a) were selected for comparison of the damage areas, which can be computed using the greyscale method of the digital image as reported in Jia’s paper [28]. The damage area calculating process was shown in Figure 11, in which the difference in areas of white color between Figure 11b,c represents the damage area of the RVE1 for BC1 configuration (Figure 11d). The ratio of damage area of the RVE1 to the area of the undamaged honeycomb cell wall was about 33.3%, while that was about 11.1% for the RVE2. Cutting damages of the BC2 configuration are less serious than that of the BC1 configuration due to the rigid body constraint on the top of the honeycomb makes the specimen stiffer. It can be concluded that bonding both top and bottom of the honeycomb to two stiffer parts individually will be beneficial to obtain good cutting quality for aramid honeycomb cutting, although it increases the cutting force. It means that cutting the honeycomb sandwich structure is a better choice than cutting the honeycomb itself in terms of cutting damage being reduced.

## 5. Conclusions

In this paper, a progressive damage modelling method based on 3D Hashin criteria was proposed to investigate the aramid honeycomb cutting process via Abaqus/Explicit with VUMAT subroutine, which can effectively predict the cutting damages and reveal the cutting mechanism. The proposed method gave a way for efficient honeycomb cutting modelling by simulating the real manufacturing process, and the material assignment problem of honeycomb in the finite element model by developing a material calculating method considering material properties of the core material and the resin. A validation experiment was conducted for ACCH-1-1.83-48 aramid honeycomb specimen. The comparison results of cutting force response and cutting damages between the finite element model and the experiment validated that the proposed method was effective for investigating the cutting process for the aramid honeycomb. Major conclusions are as follows:

(1) Cutting process of the aramid honeycomb can be divided into three stages with four characteristic states: initial state, cut-in state, cut-out state, and final state; the aramid honeycomb was cut off in the Mode I and Mode II mixed fracture mode;
(2) Cutting force acting on the low stiffness cell wall of the honeycomb in the cutting direction makes the cell wall bent, which will relieve the cutting force, and strong plasticity of the aramid fiber makes it hard to break. As a result, there will be uncut fiber and burr damages;
(3) Using sharp tip cutting tool is advantageous to obtain good cutting quality with less damages and lower cutting force;
(4) Cutting honeycomb sandwich structure directly will be beneficial to obtain good cutting quality due to the fact that skins bonded to the honeycomb make it stiffer.

## Figures and Tables

**Table 1:** The resin content for different density honeycomb [21].

**Honeycomb Density (kg/m^3^)**	48	64	80	96	123
**Resin Content (%)**	34.1	50.6	60.4	67.0	74.3

**Table 2:** Initiation criteria and schemes for seven failure types.

Failure Type	Condition	Failure Initiation Criterion	Scheme
Fiber tensile failure (FT)	$\sigma_{1}>0$	${\left(\frac{\sigma_{1}}{S_{\mathrm{LT}}}\right)}^{2}+{\left(\frac{\tau_{12}}{S_{\mathrm{LS}}}\right)}^{2}+{\left(\frac{\tau_{13}}{S_{\mathrm{LS}}}\right)}^{2}=e_{\mathrm{FT}}^{2}$	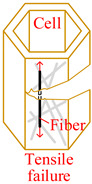
Fiber compressive failure (FC)	$\sigma_{1}<0$	${\left(\frac{\sigma_{1}}{S_{\mathrm{LC}}}\right)}^{2}=e_{\mathrm{FC}}^{2}$	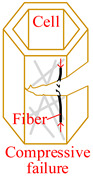
In-plane matrix cracking (MT)	$\sigma_{2}>0$	${\left(\frac{\sigma_{2}}{S_{\mathrm{HT}}}\right)}^{2}+{\left(\frac{\tau_{12}}{S_{\mathrm{LS}}}\right)}^{2}+{\left(\frac{\tau_{23}}{S_{\mathrm{HS}}}\right)}^{2}=e_{\mathrm{MT}}^{2}$	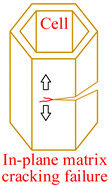
In-plane matrix crushing (MC)	$\sigma_{2}<0$	${\left(\frac{\sigma_{2}}{S_{\mathrm{HC}}}\right)}^{2}+{\left(\frac{\tau_{12}}{S_{\mathrm{LS}}}\right)}^{2}+{\left(\frac{\tau_{23}}{S_{\mathrm{HS}}}\right)}^{2}=e_{\mathrm{MC}}^{2}$	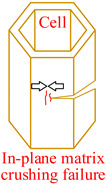
Delamination in tension (DT)	$\sigma_{3}>0$	${\left(\frac{\sigma_{3}}{S_{\mathrm{HT}}}\right)}^{2}+{\left(\frac{\tau_{13}}{S_{\mathrm{LS}}}\right)}^{2}+{\left(\frac{\tau_{23}}{S_{\mathrm{HS}}}\right)}^{2}=e_{\mathrm{DT}}^{2}$	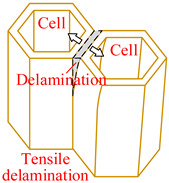
Delamination in compression (DC)	$\sigma_{3}<0$	${\left(\frac{\sigma_{3}}{S_{\mathrm{HC}}}\right)}^{2}+{\left(\frac{\tau_{13}}{S_{\mathrm{LS}}}\right)}^{2}+{\left(\frac{\tau_{23}}{S_{\mathrm{HS}}}\right)}^{2}=e_{\mathrm{DC}}^{2}$	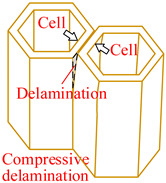
Fiber-matrix shear-out (FMS)	$\sigma_{1}<0$	${\left(\frac{\sigma_{1}}{S_{\mathrm{LC}}}\right)}^{2}+{\left(\frac{\tau_{12}}{S_{\mathrm{LS}}}\right)}^{2}+{\left(\frac{\tau_{13}}{S_{\mathrm{LS}}}\right)}^{2}=e_{\mathrm{FMS}}^{2}$	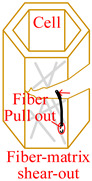

**Table 3:** Material properties of the core material [23,24,25,26], phenolic resin [13,25,27], and impregnated honeycomb.

Material Properties	Core Material	Phenolic Resin	Impregnated Honeycomb
*E*_L_/MPa	3000	4800	3614
*E*_H_/MPa	93	4800	1698
*ν*	0.193	0.389	0.26
*S*_LT_/MPa	86	45.5	72
*S*_HT_/MPa	38	45.5	40.6
*S*_LC_(*S*_HC_)/MPa	1.17	155	53.6
*S*_LS_/MPa	1.16	49	17.5
*S*_HS_/MPa	0.67	49	17.2
*ρ*/g cm^−3^	0.48	1.33	0.77

## Data Availability

All the data supporting the conclusions of the study have been provided in the paper.
